# Influence of Latent Heat of Fusion on the Melt Pool Shape and Size in the Direct Laser Deposition Process

**DOI:** 10.3390/ma15238349

**Published:** 2022-11-24

**Authors:** Gleb Turichin, Dmitrii Mukin, Ekaterina Valdaytseva, Maksim Sannikov

**Affiliations:** World-Class Research Center “Advanced Digital Technologies”, State Marine Technical University, 190121 Saint-Petersburg, Russia

**Keywords:** additive manufacturing, latent heat, phase change, enthalpy, mushy zone

## Abstract

The melt pool calculating method is presented based on the solution of the heat conduction problem in a three-dimensional formulation, taking into account the latent heat of fusion and the change in thermophysical properties with temperature. In this case, the phase transitions of melting and crystallization are accounted for using the source method. Considering the latent heat of fusion in the heat transfer process leads to melt pool elongation, as well as to a slight decrease in its width and depth. Depending on the mode, the melt pool elongation can be up to 22%. The penetration depth is reduced by about 5%. The deposition width does not change practically. The presented model was validated by comparing the experimentally determined melt pool shape and its dimensions with the corresponding theoretically calculated results. Experimental data were obtained as a result of coaxial video recording and the melt pool crystallization. The calculated form of the crystallization isotherm changes from a U-shape to a V-shape with an increase in the power and speed of the process, which coincides with the experimental data.

## 1. Introduction

Direct laser deposition (DLD) technology implies the manufacture of a physical object (part) by layer-by-layer deposition of a metallic material [1,2]. The main feature of the process is the local action of focused laser radiation on the material, which leads to the melt pool formation on the surface of the material. In this case, the filler material in the form of powder or wire is fed directly into the treatment zone or into the melt pool. DLD technology makes it possible to manufacture large-sized, non-standard products or complex-shaped parts in high-tech industries, such as aircraft construction, shipbuilding, engine construction, and so on with minimum expenses [3,4,5]. The reduction in the production cycle for the manufacture of parts is achieved due to the partial or complete exclusion of technological operations, such as casting, machining and welding. In addition, products obtained by DLD can have mechanical properties that significantly exceed ASTM standards for forged and cast material [6]. DLD technology also allows transitional layers of other metals and alloys to be made during the deposition process, which makes it suitable for product fabrication with a functional gradient transition of dissimilar materials [7,8]. These features make it possible to successfully use DLD in production, repair and restoration work.

The use of a laser as an energy source leads to the formation of a unique thermal mode, which is characterized by high cooling rates and low melt pool volumes [9]. In addition, the transient nature of heat transfer phenomena has a strong influence on the solidification rate and the temperature gradient in the deposited material. Preliminary or concomitant calculation of the temperature field and the melt pool size in the DLD process is one of the ways to maintain process stability, as well as to minimize emerging defects. Thus, in the DLD process, defects are also encountered, such as non-fusion, the main cause of which is insufficient heat source energy while simultaneously feeding an excess amount of filler material into the melt pool [10]. The incorrect choice in mode can also lead to the formation of cracking and delamination [11]. High scanning speed can cause hydrodynamic instability in the melt pool [12,13]. The above effects can be prevented by optimizing the process, which includes strict control of operating parameters, the presence or absence of preheating, control of the fusion zone and cooling rate.

Numerical and analytical modeling methods coupled with experimental data are two key approaches to process optimization. Joy Gockel, J. Beuth et al. [14,15] developed a solidification microstructure map and a melt geometry map based on finite element analysis. The authors showed that maintaining a constant cross-sectional area of the melt pool leads to a constant grain size. It is also shown that the transient effects of the scan strategy create significant variations in the melt pool geometry, which as a consequence affects the solidification condition of the material [16]. In another work [17], as a result of modeling the DLD process, the authors determined that changes in the melt pool dimensions and crystallization parameters depend on the melt pool location on the trajectory.

The melt formation in local areas has a significant effect on changes in the structure and properties of metallic materials that have undergone primary crystallization, as well as volumes located in close proximity to them. Increasing the accuracy of calculating the pool dimensions can be achieved by taking into account the melting and solidification phenomena, thereby including the latent heat of fusion in the calculation. Hu and Argyropoulos [18] discuss and compare the main methods to account for phase transformation using both analytical and numerical methods to solve the heat conduction problems. The authors state that tracking the phase transition moving front is difficult to apply in numerical methods for three-dimensional cases. An alternative and simpler approach is to reformulate the problem in such a way that the Stefan condition is implicitly included in the new form of the equations. Thus, the most widely used approach in the literature is to take into account phase transformations by modifying the specific heat capacity in such a way as to consider the latent heat of fusion [19,20,21]. De Lange et al. [22] model the effect of the latent heat of fusion on the melt isotherm shape in the two-dimensional case with a change in the temperature range, over which the latent heat was distributed into the heat capacity. Peyre et al. [23] developed a three-stage DLD model, taking into account the powder temperature and the deposited bead geometry. Before solving the heat transfer problem using finite element method, the layer height is calculated based on the law of conservation of mass and energy and the layer width is calculated by the iterative method. Cao and Ayalew [24] proposed a multi-input, multi-output control-oriented model for direct laser deposition processes. The aim of this work is to control the height of the layer and the average temperature of the melt pool. The authors noted the important role of temperature modeling in quality control of the final product.

The most adequate way of accounting for latent heat from a physical point of view is the method of sources or the enthalpy method, since both are conservative ways to introduce the latent heat [25]. Prakash et al., presented the solution of phase change problems involving a moving heat source [26]. The procedure works on a fixed grid and does not require the implementation of the Stefan condition at the solid–liquid interface. The implementation of the phase transition phenomenon for the laser welding process calculation based on the analytical approach is described by Karkhin [27]. It is shown that latent heat has a pronounced effect on the shape and size of the weld pool and the mushy zone; namely, the molten pool takes on the typical elongated and tear drop shape. In addition, the developed two-dimensional model of laser welding also takes into account the temperature dependence of the latent heat of fusion.

This work presents a method for calculating the melt pool based on the solution of the heat conduction problem in a three-dimensional formulation, taking into account the latent heat of fusion. Furthermore, the material coefficients in the heat transfer equation are approximated by a linear dependence, which makes it possible to consider the temperature dependence of the thermophysical properties of materials. The heat transfer model makes it possible to determine the melt pool size and shape, temperature fields and cooling rates of the material in the region of interest. In this case, the phase transitions in melting and crystallization are taken into account using the source method. The presented model was verified by comparing the experimentally determined melt pool shape and its dimensions with the corresponding theoretically calculated results. Moreover, this work is one of the few where an attempt was made to study and evaluate the melt pool length.

## 2. Model and Methods Description

In the presented calculation method, the following physical assumptions were made:

- The physical properties of the material such as thermal conductivity *λ*(*T*) and specific heat capacity *c*(*T*) (or volumetric heat capacity *c*_v_(*T*)) are a linear function of temperature. Density *ρ* does not depend on temperature.
- The effect of convection of liquid metal is not considered.
- The laser heat source action is described as a surface heat source.

A change in the temperature of a material leads to a change in its enthalpy. The enthalpy value *h*(*T*) can be represented as the sum of two terms, the first of which is sensible heat, which depends on temperature in a certain way, and the second is the latent heat of fusion:

(1) $$h(T)=\int_{T_{0}}^{T}c(T)dT+H(T),$$

where *c*(*T*)—specific heat capacity, *H*(*T*)—latent heat of fusion.

In this case, the nonlinear heat equation can be written as follows:

(2) $$\rho \frac{\partial h(T)}{\partial t}=\mathrm{div}\left(\lambda (T)\mathrm{grad}T\right),$$

where *ρ*(*T*)—density, *λ*(*T*)—thermal conductivity.

The initial temperature is equal to the ambient temperature:

(3) $${\left.T(x,y,z,t)\right|}_{t=0}=T_{0}.$$

Boundary conditions on the front surface of the computational domain:

(4) $$-\lambda \frac{\partial T}{\partial n}=q_{h}(x,y),$$

where *q_h_*(*x*,*y*) is the heat flux density.

Let us move to a moving coordinate system associated with a moving heat source and consider the quasi-stationary case, then calculate the derivative on the left side of the equation. The heat Equation (2) then takes the following form:

(5) $$\mathrm{div}\left(\lambda (T)\mathrm{grad}T\right)+vc(T)\rho \frac{\partial T}{\partial x}+v\rho \frac{\partial H(T)}{\partial x}=0,$$

where *v* is scanning speed.

The next step is to partially linearize the equation, given that the material properties depend linearly on temperature, but these changes are such that the thermal diffusivity remains constant:

(6) $$\lambda (T)=\lambda_{0}\left(1+mT\right)$$

(7) $$c(T)=c_{0}\left(1+mT\right)$$

(8) $$a=\frac{\lambda (T)}{\rho c(T)}=\frac{\lambda_{0}}{\rho c_{0}}$$

Then, let us use the substitution method (in this case, Kirchhoff’s substitution [28]), which makes it possible to linearize the original equation.

(9) $$\Theta (T)=f\left(T\right)=\int_{T_{0}}^{T}\frac{\lambda (T)}{\lambda_{0}}dT=T-T_{0}+\frac{m}{2}\left(T^{2}-T_{0}^{2}\right).$$

The substitution reduces the Equation (6) to the equation.

(10) $$a\Delta \Theta +v\frac{\partial \Theta }{\partial x}+\frac{v}{c_{0}}\frac{\partial H_{f}\left(\Theta \right)}{\partial x}=0,$$

where $H_{f}(\Theta )=H\left(f^{-1}(\Theta )\right)$.

Thus, an additional term arises in the heat conduction equation, which takes into account the latent heat of melting and crystallization when describing the processes of phase transition. In this case, the linear (or preliminarily linearized) equation with respect to Θ in the absence of phase transition turns into a nonlinear equation in the presence of phase transition, since the additional term $\frac{v}{c_{0}}\frac{\partial H_{f}(\Theta )}{\partial x}$ depends non-linearly on temperature.

Then, the initial and boundary conditions are rewritten as follows:

(11) $${\left.\Theta (x,y,z,t)\right|}_{t=0}=0,$$

(12) $$-\lambda_{0}\frac{\partial \Theta }{\partial n}=q_{h}(x,y).$$

According to expression (9), the desired temperature field can be obtained as

(13) $$T(x,y,z)=\frac{1}{m}\left(\sqrt{{\left(1+mT_{0}\right)}^{2}+2m\Theta (x,y,z)}-1\right).$$

**Figure 1:**
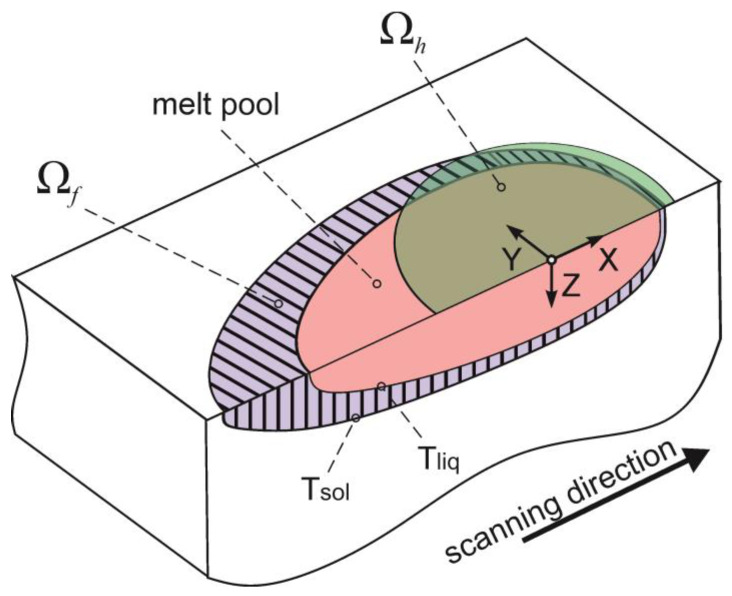
Scheme of the melt pool (green area—the heat source action, pink—the melt pool, purple—phase transformation region).

The solution to Equation (10) for a semi-infinite domain can be obtained by reducing to an integral equation using the Green’s function and the iterative method:

(14) $$\begin{array}{l} \Theta^{k}(x,y,z)=\iint_{\Omega_{h}}\frac{2q(\xi ,\eta )d\xi d\eta }{4\pi \lambda_{0}R(x,y,z,\xi ,\eta ,0)}\exp\left(-\frac{v}{2a}\left(R(x,y,z,\xi ,\eta ,0)+x-\xi \right)\right)+\\ \;\;\;\;\;\;\;\;+\sum_{i=-1,1}^{}\underset{\Omega_{f}}{∭}\frac{\partial H_{f}^{k}(\Theta^{k},\xi ,\eta ,\zeta )}{\partial x}\frac{v\rho d\xi d\eta d\zeta }{4\pi \lambda_{0}R(x,y,z,\xi ,\eta ,i\cdot \zeta )}\exp\left(-\frac{v}{2a}\left(R(x,y,z,\xi ,\eta ,i\cdot \zeta )+x-\xi \right)\right) \end{array}$$

where $R(x,y,z,\xi ,\eta ,\zeta )=\sqrt{{\left(x-\xi \right)}^{2}+{\left(y-\eta \right)}^{2}+{\left(z-\zeta \right)}^{2}}$, Ω*_h_*—heat source area, Ω*_f_*—phase transformation region (Figure 1), *k*—iteration number.

Let us use the iterative method presented in [26,27]. Then, the essence of the method is as follows:

(1) The multiplier $\frac{\partial H_{f}}{\partial x}$ is determined according to the known distribution of the field *H_f_*(Θ)^k^ at the *k* iteration;
(2) Temperature Θ^k^ is calculated according to Equation (14);
(3) Calculate *H_f_*(Θ)^k+1^ for the next iteration as $H_{f}{}^{k+1}=H_{f}{}^{k}+c_{0}\left[\Theta^{k}-\Theta (H_{f}{}^{k})\right]$ subject to the constraint 0 ≤ *H*_f_^k+1^ ≤ *L*, where Θ(*H*) is the inverse function, *L*—value of latent heat of fusion.

**Figure 2:**
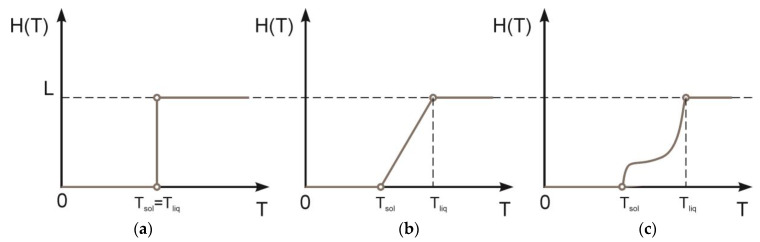
Phase change processes: (**a**) isothermal (for pure material); (**b**) linear; (**c**) general case.

The processes of melting and crystallization can be divided into two cases: the phase transition of pure substances and the phase transition of alloys. In the case of pure substances, the solid and liquid phases are separated by a sharp moving interface (Figure 2a). A phase transition occurs at a certain temperature. In alloys, the process of melting and crystallization occurs gradually and occupies the interval between the liquidus and solidus temperatures (*T_liq_* and *T_sol_*_,_ respectively). The type of curve for each alloy can be generally arbitrary (Figure 2c). Then, the latent heat of fusion *H*(*T*) can be represented as

(15) $$H(T)=\left\{\begin{array}{l} 0,\;\;\;\;\;\;\;\;\;\;\;T<T_{sol}\\ {}[0,L],\;\;\;\;T_{sol}\leq T\leq T_{liq}\\ L,\;\;\;\;\;\;\;\;\;\;T>T_{liq} \end{array}\right..$$

However, in this work, we restrict ourselves to considering only the linear dependence *H*(*T*) (Figure 2b) due to the difficulty of determining the real shape of the curve:

(16) $$H(T)=\left\{\begin{array}{l} 0,\;\;\;\;\;\;\;\;\;\;\;\;\;\;\;T<T_{sol}\\ L\frac{T-T_{sol}}{T_{liq}-T_{sol}},\;\;T_{sol}\leq T\leq T_{liq}\\ L,\;\;\;\;\;\;\;\;\;\;\;\;\;\;T>T_{liq} \end{array}\right..$$

## 3. Model Testing

Let us consider effects of latent heat of fusion on the melt pool shape and size in the DLD process of products from steel, titanium, aluminum and nickel alloys. Let us take the following technological parameters as the basic mode: laser power 2000 W, scanning speed 25 mm/s, laser spot size 2.5 mm. The material properties used for calculations are shown in Table 1.

**Figure 3:**
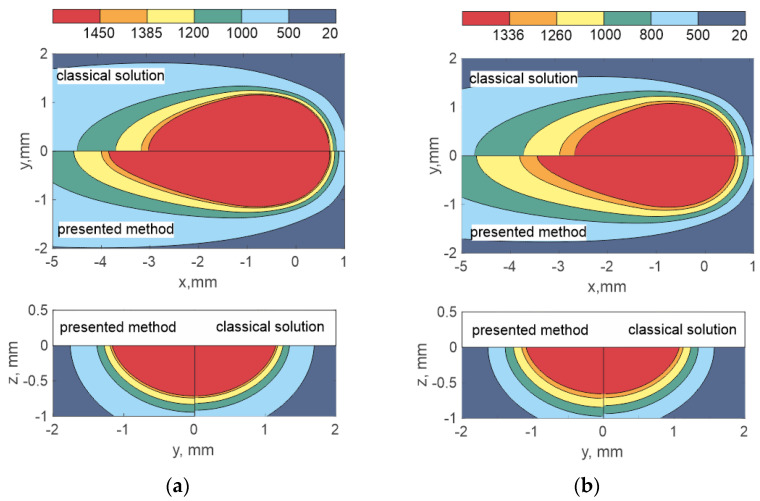
Estimated temperatures during laser deposition: (**a**) SS316L; (**b**) Inconel 718.

**Figure 4:**
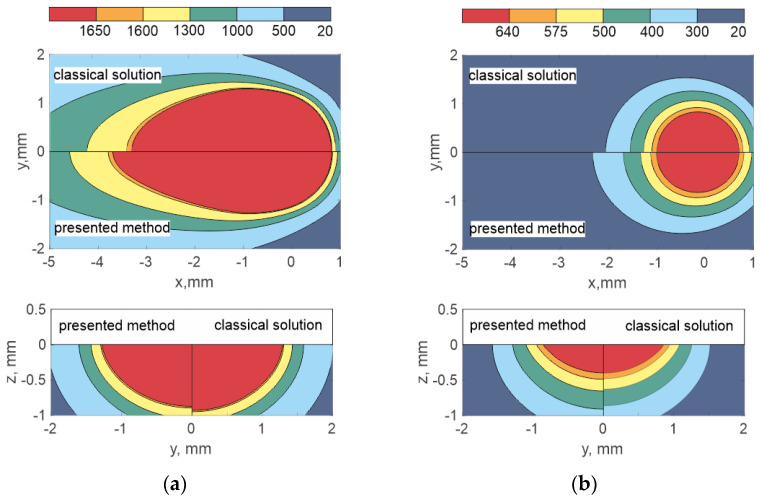
Estimated temperatures during laser deposition: (**a**) Ti6Al4V; (**b**) Al 5182.

Figure 3 and Figure 4 show a comparison of temperature fields for the cases of the classical analytical solution and the presented method, which takes into account the dependence of properties on temperature and the latent heat of fusion. The melt pool area is marked in red. The mushy zone area is orange. In the mode under consideration, the latent heat of fusion significantly lengthens the melt pool (with the exception of aluminum alloy) and does not practically affect the width and depth of penetration. In this case, the elongation degree can be completely different up to zero, as in the case of an aluminum alloy. However, it should be noted that if the function *H*(*T*) is non-linear and increases sharply towards the end of the temperature interval, it can then lead to a greater elongation of the melt pool tail than with a linear dependence [27].

**Figure 5:**
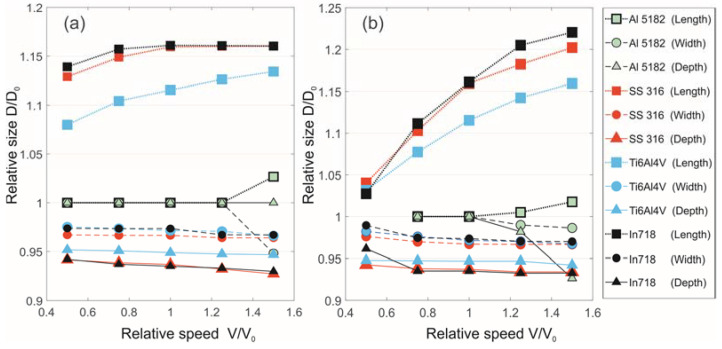
Dependence of the relative melt pool size with a change in the scanning speed (the unit is taken as the speed *V*_0_): (**a**) at constant power, (**b**) at constant linear energy.

Depending on the mode, the phase transition effect can manifest itself to different degrees. The second term in Equation (14) is responsible for the phase transformation effect, where the pre-exponential factor is directly proportional to the temperature gradient and scanning speed. In order to quantify the effect, let us vary the scanning speed at constant power and the scanning speed at variable power so that the linear energy takes a constant value. Figure 5 compares the calculation results of the current model with and without latent heat in the vicinity of the base mode (considering the second term in Equation (14) and without it). Figure 5a shows the melt pool dimensions when changing the scanning speed; the speed range varies from 0.5*V*_0_ to 1.5*V*_0_ in steps of 0.25*V*_0_, where *V*_0_ corresponds to 25 mm/s. The laser power is 2000 W. Figure 5b shows the melt pool dimensions at a constant linear energy; the change in the scanning speed is in the same range. Thus, the unit along the abscissa in both cases corresponds to the basic mode. The abscissa shows the scanning speed and the ordinate shows the relative melt pool size, where the pool size *D*_0_ (length or width or depth) is taken as a unit in the absence of phase transition.

Taking into account only the latent heat of fusion in the heat transfer process leads to the melt pool elongation, as well as to a simultaneous insignificant decrease in the penetration width and depth. Depending on the mode, the pool elongation can be up to 22%. The penetration depth is reduced by approximately 5%. The deposition width does not practically change, since during DLD the pool width is mainly determined by the mode and laser spot size on the treatment zone surface.

The latent heat of fusion of aluminum alloys takes the greatest value and occupies the largest share in the specific energy required for melting relative to other materials. Despite this, considering the phase transition does not affect the pool shape and dimensions in a wide range of modes. This is due to the fact that in the modes under consideration, the melt pool has a small size and a round shape. In order for the effect to be observed, it is necessary to increase the laser power, as, for example, in the welding process. Thus, the behavior of aluminum alloy is qualitatively different from other materials, while SS316L, Ti6Al4V and IN718 behave in a similar way. The reason for this lies in the difference in thermophysical properties between these materials. For example, the thermal diffusivity of SS316L, Ti6Al4V, and IN718 is an order of magnitude different from the thermal diffusivity of aluminum alloy. As mentioned above, the change in width and depth for SS316L, Ti6Al4V, and IN718 alloys is weakly dependent on the mode within the studied range of technological parameters. The values of the melt pool length take on a completely different character. The effect of latent heat increases slightly with increasing scanning speed and constant laser power, elongating the melt pool to a greater extent. However, in this case, the growth of the elongation function may stop and take a stationary value (see Figure 5a). At a constant heat input, the elongation takes on a pronounced increase with an increase in the scanning speed over the entire range. The greatest effect of latent heat of fusion is observed on SS316L and IN718, since both the elongation growth rate and the elongation value itself take the largest value (see Figure 5b).

Although the deposition width is reduced by about 5%, the melt pool takes on a tear drop shape, tapering sharply towards its tail. This means a change in the crystallization isotherm position, which can lead to a change in the grain growth orientation. In general, the greatest effect is observed in modes with high speeds and high powers. Conversely, the phase transition does not affect the pool dimensions when the melt pool is round and non-elongated.

## 4. Materials and Experimental Procedure

**Figure 6:**
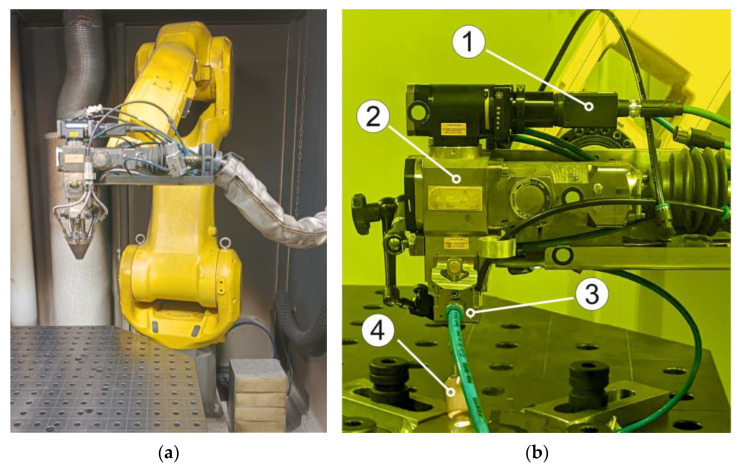
DLD complex: (**a**) with filler powder nozzle, (**b**) without filler powder nozzle. 1—coaxial camera, 2—laser head, 3—cross-jet, 4—shielding gas nozzle.

In this study, a robotic DLD complex based on an IPG fiber laser was used. The complex included a six-axis robot and a two-axis positioner manufactured by Fanuc. The laser system had a maximum power of 3000 W. The deposition process was carried out with a local supply of protective gas. Argon was used as a protective gas. A Lucid TRI032S-CC machine vision camera was installed coaxial to the laser beam to image the melt pool during processing (Figure 6). The filler powder nozzle has been changed to a cross-jet, due to the fact that the nozzle covers the melt pool tail.

Single linear tracks with lengths of 150 mm were deposited on a stainless steel 316L substrate without filler powder supply. The substrate size was 200 × 100 × 10 mm (length × width × height). Substrate temperature was measured before each track was deposited to ensure that conditions were uniform and heat accumulation did not affect the results. The substrate temperature was (50 ± 3) °C immediately before the deposition of the next track. The tracks were deposited with enough distance separation so that previous tracks did not interfere with further deposition.

All passes were carried out in one direction, the shielding gas was set to 15 L/min, the laser spot size was 3 mm, and the beam axis was set perpendicular to the substrate. In the experiment, the laser power and scanning speed were changed. The other parameters remained unchanged. Four laser power settings of 800, 1000, 2000, 2500 W and three scanning speeds of 10, 20, 30 mm/s were used. As a result, 12 combinations of technological parameters were obtained. To fix the melt pool edges, the brightness of the light falling on the camera was reduced by hardware on the laser head, and the exposure time on the coaxial camera was chosen to be 150 μs.

## 5. Results and Discussions

Since the laser beam stopped the action at the trajectory end and the robot continued to move in the original direction without changing the speed, it was assumed that during the crystallization process, the melt pool would retain its state at the moment of switching off the laser radiation. Thus, in the experiment, the end of the track was fixed in order to further measure the geometric parameters of the melt pool.

**Figure 7:**
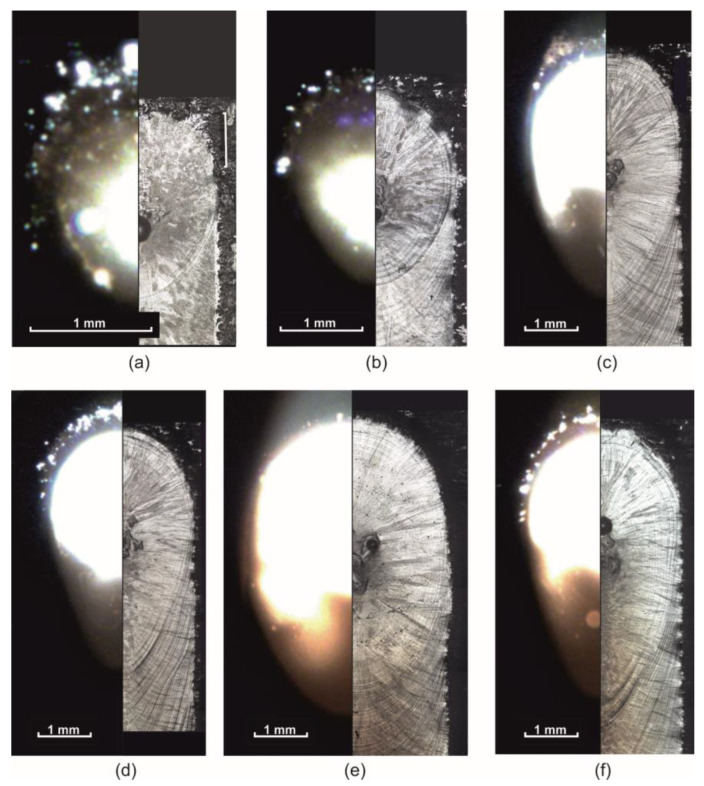
A series of melt pool images for various mode parameters taken using a coaxial camera (left) and the result of crystallization (right). Mode parameters: (**a**) 800 W, 20 mm/s; (**b**) 1000 W, 20 mm/s; (**c**) 2000 W, 20 mm/s; (**d**) 2000 W, 30 mm/s; (**e**) 2500 W, 10 mm/s and (**f**) 2500 W, 30 mm/s.

Figure 7 shows examples of melt pool images taken with a coaxial camera during processing and crystallized pools at the track end. The comparison shows the conformity of the geometric dimensions and pool shape features during video recording and as a result of crystallization. Based on this, the following can be concluded: Firstly, coaxial video recording registered a melt pool, not artifacts caused, for example, by strong illumination. Secondly, the melt pool crystallized in the state it was in at the time of switching off the laser radiation. Thirdly, the pool obtained by crystallization at the end of the track corresponds to a steady state melt pool over the entire trajectory interval.

**Figure 8:**
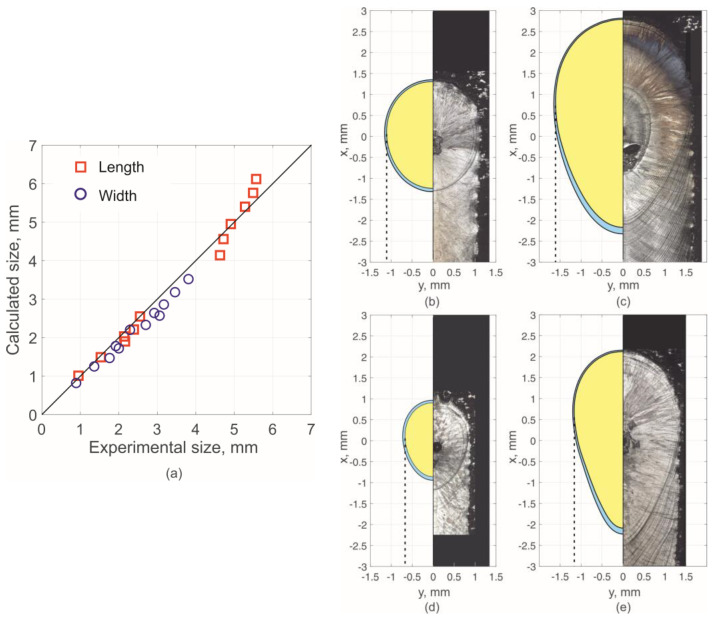
Experimentally measured dimensions of the melt pool and the corresponding calculated values: (**a**) comparison of expected and calculated values. Calculated (left) and experimental (right) form at: (**b**) 1000 W and 10 mm/s; (**c**) 2000 W and 10 mm/s; (**d**) 1000 W and 30 mm/s; (**e**) 2000 W and 30 mm/s. The yellow area corresponds to *T* > *T*_liq_, the blue area corresponds to *T*_sol_ < *T* < *T*_liq_.

Figure 8 shows a comparison of the results of experimental pool size data and calculated data using the proposed method. The calculated melt pool dimensions and shape features qualitatively agree with the experimental data. As expected, the melt pool is round at minimum power. In this case, considering the pheasant transition does not have a significant effect, as mentioned above. Such a result is observed, for example, in modes with a power of 800 and 1000 W. At 2500 W and 10 mm/s, the melt pool also has a U-shape (see Figure 7e). The greater the power and scanning speed, the greater the phase transition effect and the more the melt pool shape changes by taking on a V-shape (see Figure 7c,d,f).

The maximum value of the relative deviation in the calculated values is modulo 12% for the length and 16% for the width. In this case, the relative deviation in the calculated pool width values always takes negative values. Such a deviation can be attributed to a systematic one. This feature is possibly due to the lack of accurate data on the heat flux density distribution of laser radiation, if not related to the assumptions made. However, the calculation result remains adequate over the entire range of mode parameters.

## 6. Conclusions

This paper presents a method for calculating the melt pool based on the solution of the heat conduction problem in a three-dimensional formulation, taking into account the latent heat of fusion. In addition, the material coefficients in the heat transfer equation are approximated by a linear dependence, which makes it possible to consider the temperature dependence of the thermophysical properties of materials.

Ignoring the latent heat of fusion can significantly reduce the melt pool length, as well as slightly increase the width and depth. Thus, at the studied parameters, taking into account the phase transformation, the elongation of the melt pool goes up to 22%, and the penetration depth decreases by about 5%. The pool width does not change much. In general, the greatest effect is observed in modes with high speeds and high powers. At the same time, stainless steel 316L and Inconel 718 have the highest sensitivity in calculations. Conversely, the phase transition does not affect the pool size when the melt pool is round and not elongated. This is especially true for aluminum alloy.

The model was validated by comparing the experimentally determined shape of the melt pool and its dimensions with the corresponding theoretically calculated results. Experimental data were obtained as a result of coaxial video recording and as a result of melt pool crystallization. The proposed calculation method shows an adequate result in a wide range of mode parameters. Further research will be aimed at determining the size and shape of the melt pool whilst taking into account the effects of convective heat transfer, comparing the results of only a thermal problem (as in this case) and a related problem, and studying the possibility of using these models to predict the microstructure formation.

## Figures and Tables

**Table 1:** Material properties for SS316L, Ti6Al4V, Inconel 718, Al 5182 [29,30,31,32].

Parameter	Material			
	SS316L	Ti6Al4V	Inconel 718	Al 5182
Density (kg·m^−3^)	7400	4200	7800	2550
Thermal conductivity (W·m^−1^·K^−1^)	21 × (1 + 3×10^−4^ × T)	20.3 × (1 + 1.5×10^−4^ × T)	17.8 × (1 + 3.8×10^−4^ × T)	108 × (1 + 5×10^−4^ × T)
Specific heat capacity (J·kg^−1^·K^−1^)	515 × (1 + 3×10^−4^ × T)	609 × (1 + 1.5×10^−4^ × T)	461 × (1 + 3.8×10^−4^ × T)	924 × (1 + 5×10^−4^ × T)
Solidus temperature (°C)	1385	1600	1260	575
Liquidus temperature (°C)	1450	1650	1336	640
Latent heat of fusion (kJ·kg^−1^)	260	286	210	358
Absorption coefficient	0.44	0.43	0.33	0.23

## Data Availability

Not applicable.
